# Machine learning model for the prediction of gram-positive and gram-negative bacterial bloodstream infection based on routine laboratory parameters

**DOI:** 10.1186/s12879-023-08602-4

**Published:** 2023-10-10

**Authors:** Fan Zhang, Hao Wang, Liyu Liu, Teng Su, Bing Ji

**Affiliations:** 1 Department of Critical Care Medicine, Qilu Hospital of Shandong University, Jinan, 250012 Shandong China; 2 School of Control Science and Engineering, Shandong University, Jinan, 250061 Shandong China

**Keywords:** Bacteremia, Machine learning, Gram-positive, Gram-negative

## Abstract

**Background:**

Bacterial bloodstream infection is responsible for the majority of cases of sepsis and septic shock. Early recognition of the causative pathogen is pivotal for administration of adequate empiric antibiotic therapy and for the survival of the patients. In this study, we developed a feasible machine learning (ML) model to predict gram-positive and gram-negative bacteremia based on routine laboratory parameters.

**Methods:**

Data for 2118 patients with bacteremia were obtained from the Medical Information Mart for Intensive Care dataset. Patients were randomly split into the training set and test set by stratified sampling, and 374 routine laboratory blood test variables were retrieved. Variables with missing values in more than 40% of the patients were excluded. Pearson correlation test was employed to eliminate redundant features. Five ML algorithms were used to build the model based on the selected features. Additionally, 132 patients with bacteremia who were treated at Qilu Hospital of Shandong University were included in an independent test cohort to evaluate the model.

**Results:**

After feature selection, 32 variables remained. All the five ML algorithms performed well in terms of discriminating between gram-positive and gram-negative bacteremia, but the performance of convolutional neural network (CNN) and random forest (RF) were better than other three algorithms. Consider of the interpretability of models, RF was chosen for further test (ROC-AUC = 0.768; 95%CI = 0.715–0.798, with a sensitivity of 75.20% and a specificity of 63.79%). To expand the application of the model, a decision tree (DT) was built utilizing the major variables, and it achieved an AUC of 0.679 (95%CI = 0.632–0.723), a sensitivity of 66%, and a specificity of 67.82% in the test cohort. When tested in the Qilu Hospital cohort, the ROC-AUC of the RF and DT models were 0.666 (95%CI = 0.579–0.746) and 0.615 (95%CI = 0.526–0.698), respectively. Finally, a software was developed to make the RF- and DT-based prediction models easily accessible.

**Conclusion:**

The present ML-based models could effectively discriminate between gram-positive and gram-negative bacteremia based on routine laboratory blood test results. This simple model would be beneficial in terms of guiding timely antibiotic selection and administration in critically ill patients with bacteremia before their pathogen test results are available.

**Supplementary Information:**

The online version contains supplementary material available at 10.1186/s12879-023-08602-4.

## Background

Bacterial bloodstream infection is responsible for the majority cases of community-acquired and hospital-acquired sepsis and septic shock [1]. It is associated with poor outcomes, especially in cases where patients do not receive appropriate and timely antimicrobial therapy [2–4]. Early adequate empirical antibiotic therapy is pivotal for patients’ survival [5]. However, increasing antibiotic resistance to commonly used antimicrobials poses a challenge to treatment. Early identification of the causative pathogen is important because this can enable physicians to choose appropriate antibiotic agents for therapy. Blood culture still serves as the gold standard for identification of the causative microorganism, but it is time-consuming and shows a high false-negative rate. Other rapid diagnostic tools for the early optimization of antimicrobial therapy, such as PCR-based tests, are limited by the number of PCR probes required or are dependent on positive culture samples [1].

The causative pathogen can be identified based on the levels of specific infectious biomarkers or inflammation cytokines, such as procalcitonin, interleukin (IL)-2, IL-4, IL-6, tumor necrosis factor-α, and interferon-γ [6–9], but these parameters are not commonly measured at primary-level hospitals or hospitals in low-income countries [10]. Routine laboratory parameters, including complete blood cell (CBC) counts, acute-phase proteins, electrolytes, and blood gas indicators, are commonly measured, and the data for these variables can be easily obtained from hospitals at different levels across different countries. Using these parameters to predict the causative pathogen may present a more practical, feasible, and time-saving strategy, especially for patients who are severely ill or admitted to lower-level hospitals.

Machine learning (ML) techniques have shown great potential in aiding the diagnosis of disease [11, 12]. In recent years, ML technologies have seen remarkable advancements and are being rapidly implemented in various medical fields. A series of ML-based models have been developed successfully and have demonstrated the feasibility and interpretability of ML in bacteremia prediction [13–16]. Beeler et al. [13] developed a model using the random forest (RF) algorithm to predict the risk of central line-associated bloodstream infections (CLABSIs). Further, Mahmoud et al. [14] used six ML algorithms, including RF, logistic regression (LR), decision trees (DT), naive Bayes (NB), artificial neural networks (ANN), and support vector machine (SVM), to discriminate between positive and negative blood cultures with high specificity. Tsai et al. [15] established models based on LR and SVM to predict bacteremia in febrile children. In another such study, Bhavani et al. [16] demonstrated that ML models based on data from electronic health records exhibited better performance in predicting bacteremia than previously published risk scores.

As far as we know, routine laboratory parameters have not yet been successfully integrated in an ML model for predicting gram-positive and gram-negative infections in patients with bacteremia. Therefore, in this study, we aimed to establish an ML model for early identification of gram-positive and gram-negative bacteremia based on routine laboratory parameters. 1–3-β-d-glucan test is a good diagnosis tool of invasive fungal infection with great sensitivity and specificity, so the BSI patients caused by fungus were not included in this research. An ML-based model utilizing the LR, SVM, ANN, RF, and convolutional neural network (CNN) algorithms was constructed and evaluated in the Medical Information Mart for Intensive Care (MIMIC) cohort (from the USA) and a local cohort (from China) in order to provide robust evidence for the application of this model.

## Methods

### Study design

**Fig. 1:**
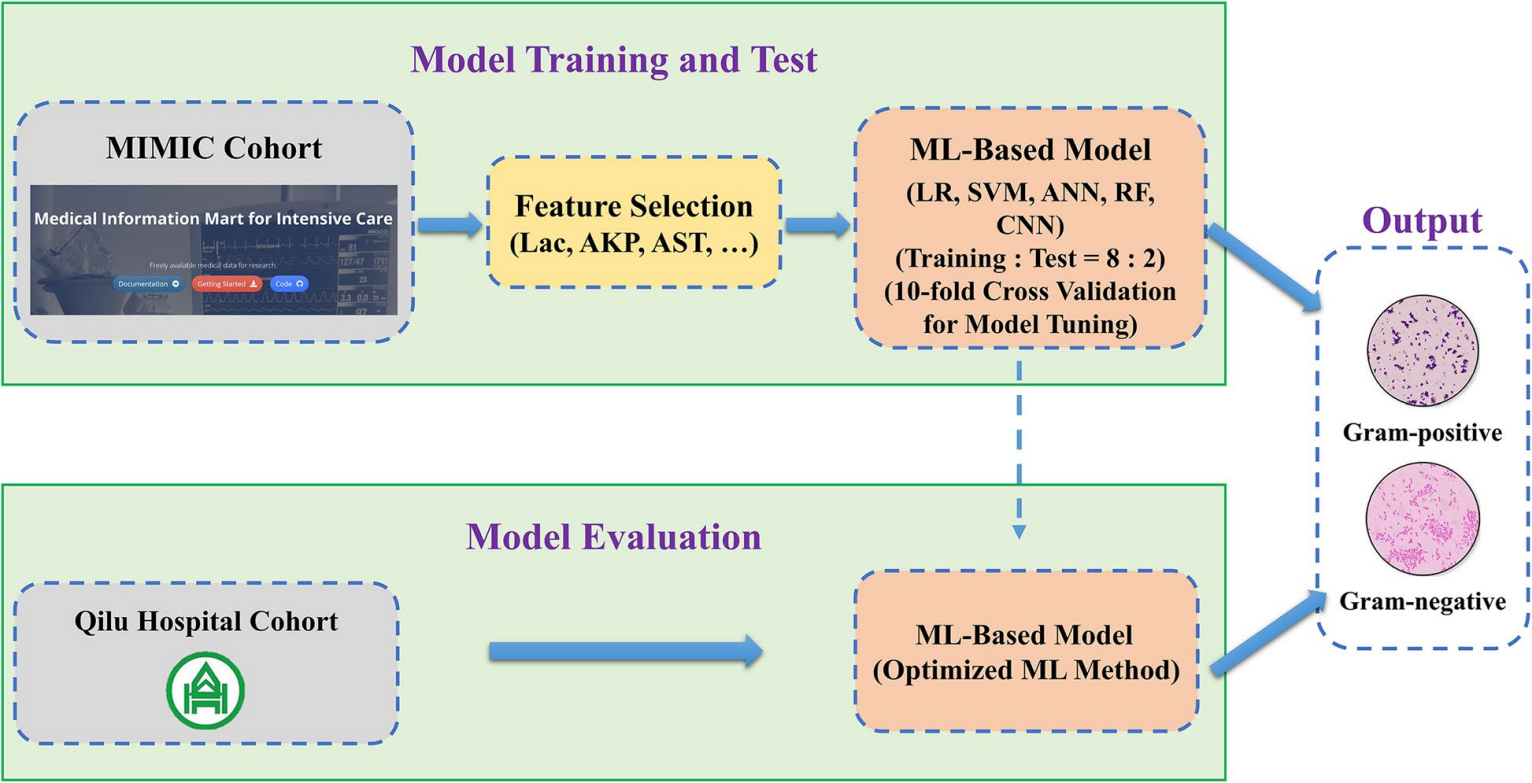
Schematic depiction of the study design

As shown in Fig. 1, the MIMIC cohort was divided into a training set and a test set. Routine laboratory blood test results were extracted from the MIMIC dataset. Parameters with large amounts of data missing were removed, and the Pearson correlation test was used to remove redundant features. After feature selection, five ML algorithms (LR, SVM, ANN, RF, and CNN) were used to build the model based on the selected features. The built model was then evaluated on a hold-out test set of the MIMIC cohort. In addition, data collected from an independent test cohort from Qilu Hospital of Shandong University were used to evaluate the model further.

### Source of data and study population

MIMIC is an open critical care database that contains comprehensive clinical data of patients admitted to Beth Israel Deaconess Medical Center in Boston, Massachusetts [17–19]. MIMIC III contains data collected between June 2001 and October 2012, while data collected between 2008 and 2018 are recorded in MIMIC IV. The datasets used in the present study were extracted by Zhang and Wang, who have completed the collaborative institution training initiative program course (Record ID: 36181465 and 46463103).

**Fig. 2:**
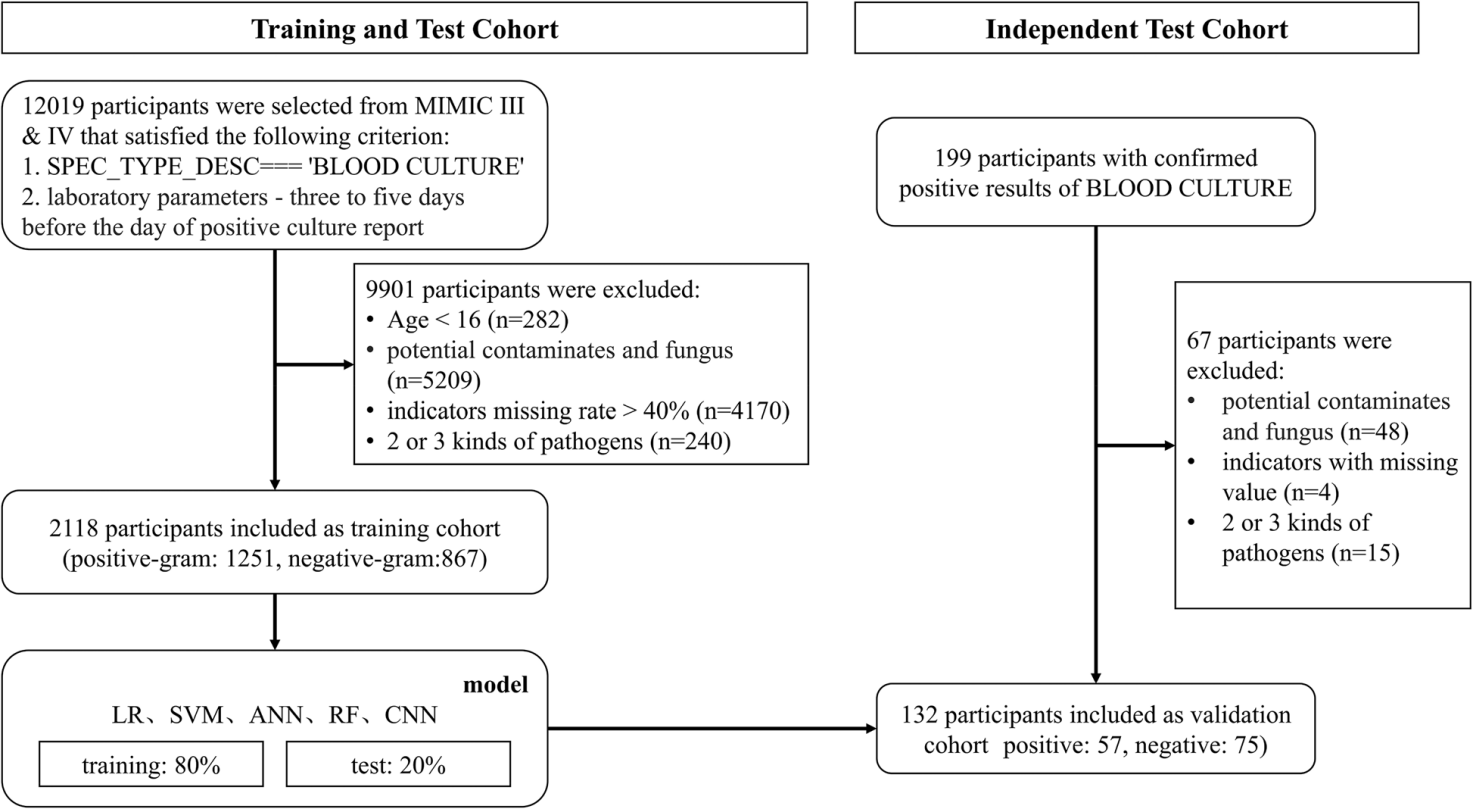
Flowchart depicting the cohort selection process

As shown in Fig. 2, data for patients with a positive blood culture for a bacterial pathogen were retrieved. The exclusion criteria were age less than 16 years and the detection of microorganisms that were potentially contaminants, as pre-defined according to previous reports [20, 21]. For patients with multiple episodes of bacteremia, only data for the first episode were retrieved. Moreover, cases of mixed infection with two or three microorganisms detected in the same blood sample were also excluded. We retrieved laboratory results for blood examinations that were conducted closest to the onset of the bacteremia (defined as the collection time of the positive blood sample). Parameters for which data were missing in more than 40% of the patients were excluded.

Another cohort from outside of the USA was used to evaluate the model further. This cohort was recruited from two mixed ICUs in Qilu Hospital of Shandong University in China. Patients with positive blood bacterial culture between 2019 and 2020 were included according to a protocol approved by the Ethics Committee of Qilu Hospital, Shandong University (approval no. KYLL-2018153). Written informed consent was waived by the ethics committees because the study did not involve any interventions.

### Variables

Initially, 374 variables were obtained from routine laboratory blood tests, including CBC count, liver function test, renal function test, serum cardiac markers, serum electrolytes, blood gas analysis, coagulation-associated markers, and immune cell-associated markers. However, 340 variables for which data were missing in > 40% of the patients were eliminated, and the remaining 34 variables were utilized for development of the model. All these 34 variables can be measured by routine laboratory blood tests for emergency or critical patients, and they include CBC count, liver function test, renal function test, serum electrolytes, blood gas analysis, and coagulation function test.

### Statistical analysis

The Mann–Whitney *U*-test was utilized to analyze differences between gram-positive and gram-negative cases in terms of numerical features. Pearson correlation test was utilized to assess the linear dependence between features. Redundant features (Pearson correlation coefficient $\left|\gamma \right|\ge 0.8$) were removed [22] to ensure that there was no high degree of correlation in the final selected features for the model. The Delong test was performed to compare the area under the curve (AUC) of the classifiers used to construct the model. *P* < 0.05 was considered to indicate statistical significance for all the analyses. Statistical analysis was performed using Python in Pycharm-Professional-2019.1.3.

### Model development

The model was built based on five classifiers: LR, SVM with the radial basis function kernel, ANN, RF, and CNN. As LR, SVM, and ANN are sensitive to the dimension of features, Z-score analysis was performed before model training. Receiver operating characteristic (ROC) curve analysis was used to evaluate the performance of the built model, and AUC was computed. Accuracy, sensitivity, specificity, positive predictive value (PPV), and negative predictive value (NPV) were also calculated to assess the performance of the model.

In order to determine the importance of each feature, SHapley Additive explanation (SHAP) values [23] were computed based on each model. With SHAP, an additive interpretation model can be constructed in which all the features are regarded as contributors, and then the marginal contribution of a feature can be calculated for each sample when it is added to the model. Since a feature has different marginal contributions for different feature sequences, the mean value is computed as the SHAP value. Eventually, the mean of all the samples’ SHAP values for a feature was considered to indicate the feature’s importance. Furthermore, considering that all 34 variables used in the model may not always be available, a predictive decision tree (DT) model requiring fewer parameters was also developed for the differentiation of gram-positive and gram-negative bacteria.

The MIMIC cohort was randomly partitioned into the training set and the test set at a ratio of 8:2, and the training and test sets had the same distribution of gram-positive and gram-negative bacteremia cases as the original dataset. This process was repeated five times to diminish the influence of data partitioning. Model tuning was performed with ten-fold cross validation, and the tuned model was tested on the test set. The median performance after five rounds of testing was finally reported in our study. The entire process was performed in Pycharm-Professional-2019.1.3. We use scikit-learn library of 1.2.2 version and pytorch library of 1.11.0 version.

## Results

### Patient characteristics and variables

In total, 2118 patients whose records were deposited in the MIMIC-III and MIMIC-IV database were enrolled; this included 1251 patients with gram-positive and 867 patients with gram-negative bacteremia. The three most common pathogens identified in the gram-positive group were *Staphylococcus aureu*s (*n* = 599), *Enterococcus faecium* (*n* = 252), and *Enterococcus faecalis* (*n* = 106), and the three most common gram-negative bacteria were *Escherichia coli* (*n* = 347), *Klebsiella pneumoniae* (*n* = 163), and *Pseudomonas aeruginosa* (*n* = 70). The median age of the two groups was comparable, and 61.19% (*n* = 1296) of the patients were male and 38.81% (*n* = 822) were female. We excluded variables with missing values exceeding 40%, and then we selected patients who did not have missing variables.

**Fig. 3:**
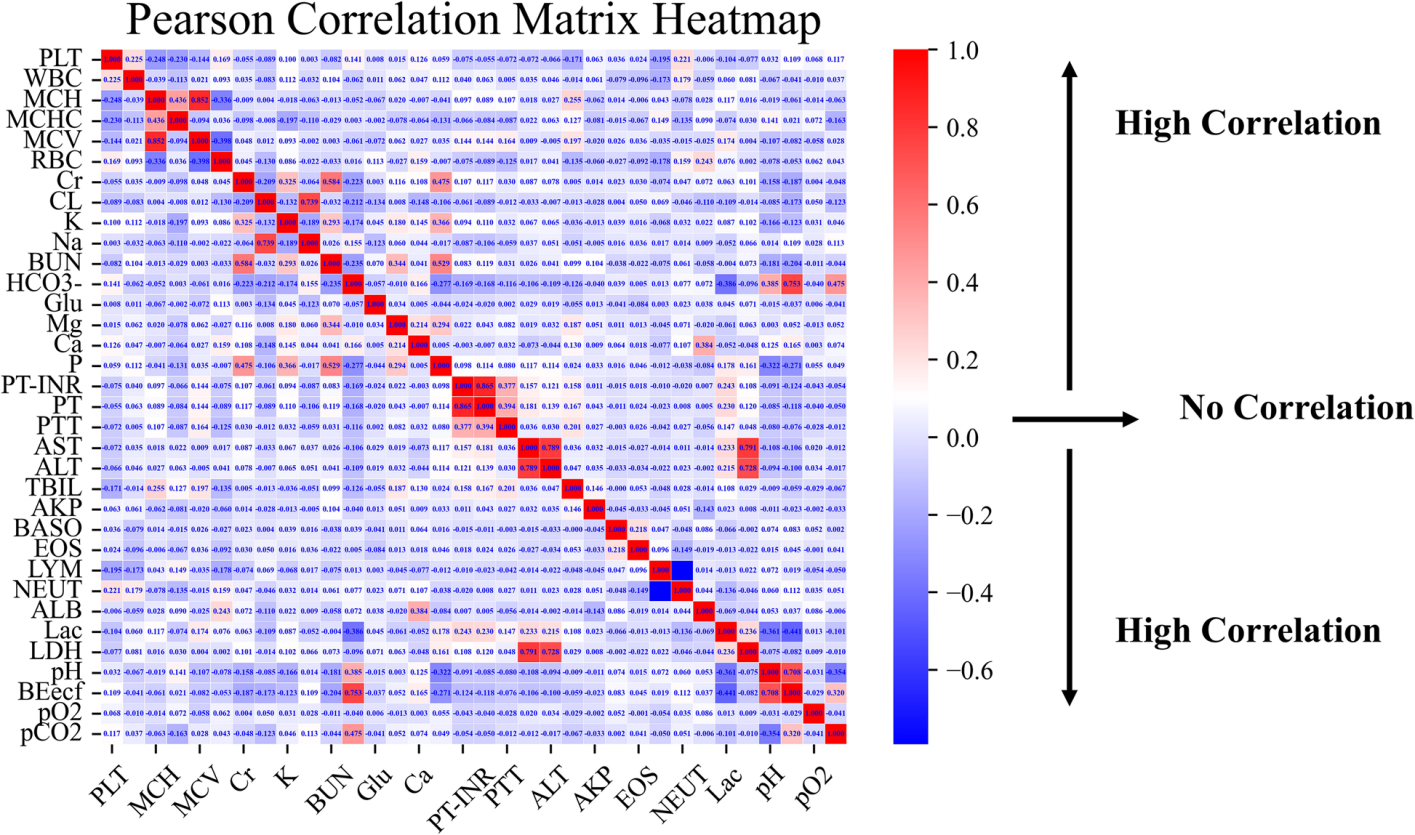
Heatmap of Pearson correlation coefficients for the correlations among variables. A high absolute value of the Pearson correlation coefficient corresponds to a high degree of correlation. The dark red and dark blue squares indicate a high degree of correlation, while the light red and light blue squares indicate a low degree of correlation

Figure 3 shows a heatmap of the correlation coefficients for the correlations between the variables. Variables with a high degree of correlation were eliminated, and 32 variables were eventually selected for input into the model. All 32 variables can be measured with routine blood tests in the emergency department or ICU. Table 1 shows patients’ characteristics, including age, gender, and the 32 selected variables.

**Table 1:** Distribution of patients’ demographics characteristics and routine laboratory parameters

	**Variables**	**Total**	**Gram-negative**	**Gram-positive**	***P* value**
	No. of patients(%)	2118(100)	867(40.93)	1251(59.07)	
1	Age, median(Q1-Q3), year	63(53–74)	63(52–73)	64(53–75)	0.380
2	Gender, No. (%)				0.388
	male	1296(100)	521(40.20)	775(59.80)	
	female	822(100)	346(42.09)	476(57.91)	
3	PLT, mean(SD), K/μL	194.82(141.88)	191.42(142.50)	197.17(141.45)	0.359
4	WBC, mean(SD), K/μL	13.02(10.81)	12.62(11.41)	13.30(10.37)	0.159
5	BASO, mean(SD), %	0.19(0.35)	0.15(0.29)	0.22(0.38)	< 0.0001*
6	EOS, mean(SD), %	0.90(2.07)	0.81(1.77)	0.97(2.25)	0.089
7	LYM, mean(SD), %	12.25(16.75)	12.11(16.61)	12.34(16.85)	0.760
8	NEU, mean(SD), %	75.39(20.92)	73.53(21.53)	76.68(20.40)	0.001*
9	RBC, mean(SD), m/μL	3.40(0.72)	3.36(0.71)	3.43(0.73)	0.027*
10	MCHC, mean(SD), %	33.06(1.69)	33.07(1.65)	33.05(1.72)	0.776
11	MCV, mean(SD), fL	91.49(8.02)	92.50(8.13)	90.79(7.87)	< 0.0001*
12	AST, mean(SD), IU/L	188.77(1021.59)	205.59(820.69)	177.12(1140.43)	0.528
13	ALT, mean(SD), IU/L	112.96(435.01)	128.28(449.60)	102.35(424.47)	0.177
14	TBIL, mean(SD), mg/dL	3.05(6.21)	3.89(7.05)	2.47(5.48)	< 0.0001*
15	AKP, mean(SD), IU/L	171.32(196.65)	210.26(241.01)	144.32(153.20)	< 0.0001*
16	ALB, mean(SD), g/dL	2.81(0.64)	2.79(0.64)	2.82(0.63)	0.171
17	LDH, mean(SD), IU/L	463.58(1032.98)	478.21(1108.49)	453.43(977.56)	0.587
18	Cr, mean(SD), mg/dL	2.01(2.06)	1.96(1.75)	2.04(2.25)	0.366
19	BUN, mean(SD), mg/dL	37.25(28.33)	38.17(29.15)	36.62(27.74)	0.215
20	Chloride, mean(SD), mEq/L	102.02(7.18)	102.01(6.91)	102.02(7.36)	0.966
21	Potassium, mean(SD), mEq/L	4.19(0.81)	4.17(0.85)	4.20(0.78)	0.281
22	Sodium, mean(SD), mEq/L	137.24(5.85)	137.09(5.49)	137.34(6.09)	0.322
23	Magnesium, mean(SD), mg/dL	1.96(0.42)	1.93(0.46)	1.98(0.39)	0.006*
24	Calcium, mean(SD), mg/dL	8.25(0.95)	8.21(0.99)	8.28(0.92)	0.106
25	Phosphate, mean(SD), mg/dL	3.58(1.62)	3.57(1.69)	3.59(1.57)	0.810
26	Bicarbonate, mean(SD), mEq/L	22.58(5.45)	21.76(5.73)	23.15(5.17)	< 0.0001*
27	Glu, mean(SD), mg/dL	142.42(72.63)	140.37(76.69)	143.83(69.68)	0.281
28	pH, mean(SD), units	7.38(0.10)	7.37(0.11)	7.39(0.09)	< 0.0001*
29	BEecf, mean(SD), mEq/L	-1.80(5.68)	-2.95(6.32)	-1.01(5.05)	< 0.0001*
30	pO2, mean(SD), mm Hg	11.58(77.79)	108.36(74.76)	113.80(79.78)	0.114
31	pCO2, mean(SD), mm Hg	38.66(10.79)	37.82(10.42)	39.24(11.00)	0.003*
32	Lac, mean(SD), mmol/L	2.65(2.24)	3.31(2.75)	2.20(1.66)	< 0.0001*
33	PT-INR, mean(SD)	1.70(1.10)	1.79(1.31)	1.63(0.93)	0.003*
34	PTT, mean(SD), sec	38.28(19.57)	39.71(19.78)	37.28(17.63)	0.004*

Absolute numbers and percentages are used for categorical variables and mean and standard deviation are used for continuous variables

^*^shows the significant differences between the gram-positive and gram-negative

### Construction of the predictive model for differentiating between gram-positive and gram-negative bacteremia

**Fig. 4:**
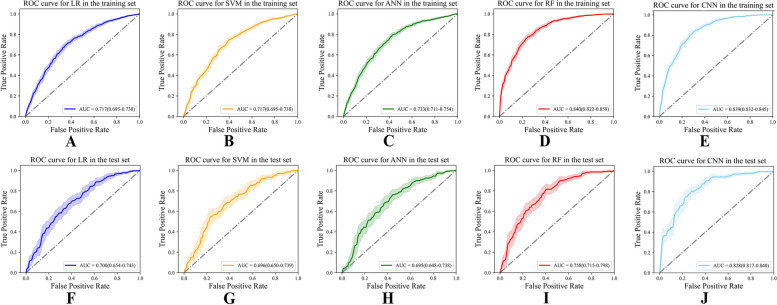
ROC curves depicting the predictive ability of the model in the training set and test set

The predictive model was constructed based on four ML classifiers, i.e., LR, SVM, ANN, RF, and CNN. Figure 4 presents the ROC curves of the model based on the five classifiers for the training set and the test set. As demonstrated in Fig. 4, in both sets, RF performed better (AUC = 0.768, 95% CI = 0.715–0.798) than LR, SVM, and ANN (*p* = 0.0011, *p* = 0.0001, and *p* = 0.0039, respectively) with regard to discriminating between gram-positive and gram-negative bacteremia, while the other three classifiers had comparable performances (*p* > 0.05). We also compared the five-fold cross-validation results with ten-fold cross-validation based on RF according to your suggestions. It is found that the ten-fold cross-validation performed better (AUC = 0.768, 95% CI = 0.715–0.798) than five-fold cross-validation (AUC = 0.762, 95% CI = 0.708–0.796).The performance of CNN (AUC = 0.828, 95% CI = 0.817–0.840) was slightly better than RF (*p* = 0.0043). However, considering the CNN model lacking interpretability, which is very important in clinical application, the RF model was finally chosen for further research analysis.

**Fig. 5:**
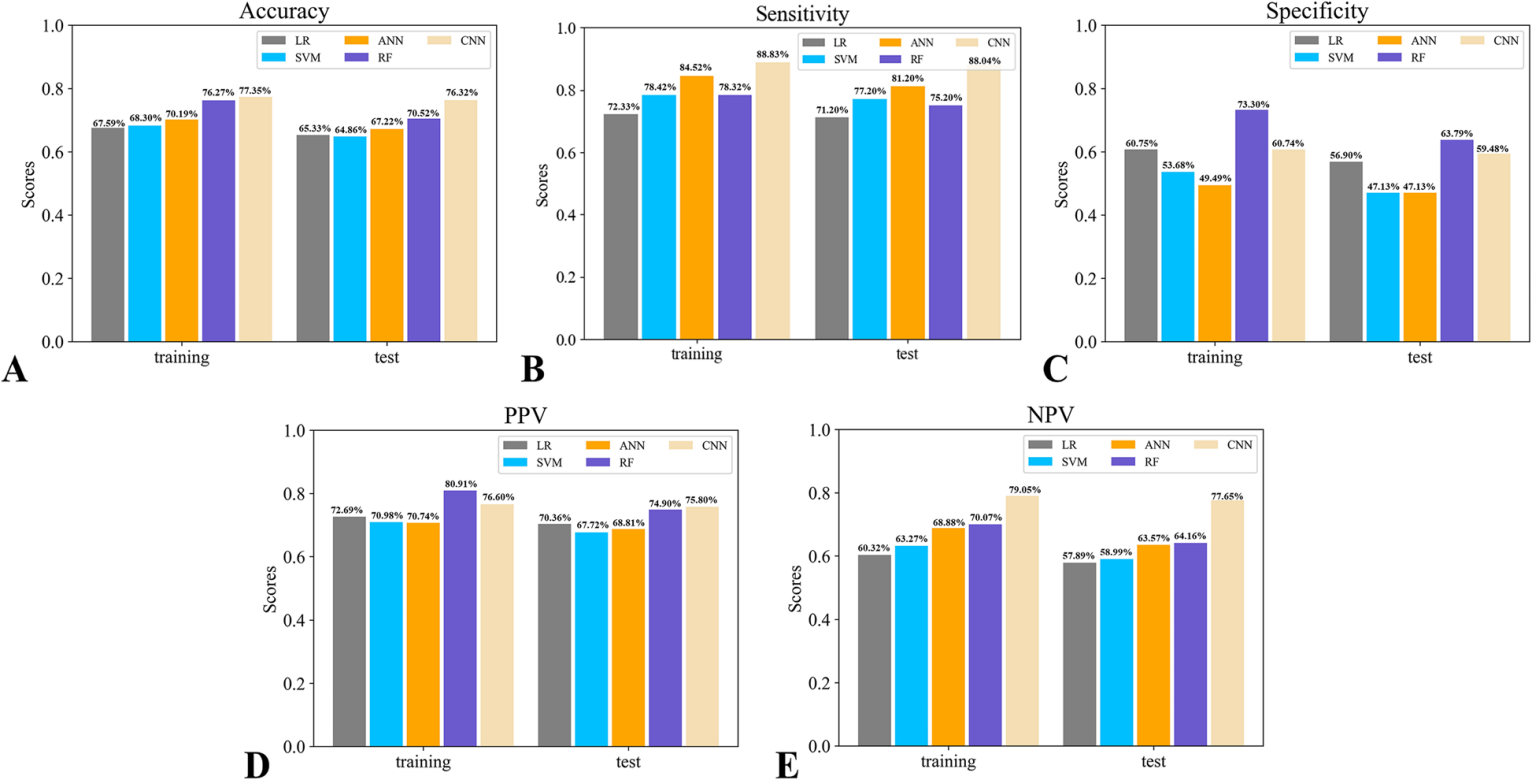
Performance of the models based on their accuracy (**A**), sensitivity (**B**), specificity (**C**), PPV (**D**), and NPV (**E**)

Figure 5 shows the performance of the model in the training and test sets based on accuracy, sensitivity, specificity, PPV, and NPV. As shown in Fig. 5, RF achieved higher accuracy, specificity, PPV, and NPV than the other ML algorithms. Although both SVM and ANN had higher sensitivity than RF, the sensitivity of RF was still high at 75.20% in the test set and was effective for distinguishing between gram-positive and gram-negative bacteremia. Thus, the model was eventually built based on the RF classifier to predict gram-positive and gram-negative bacteremia.

**Fig. 6:**
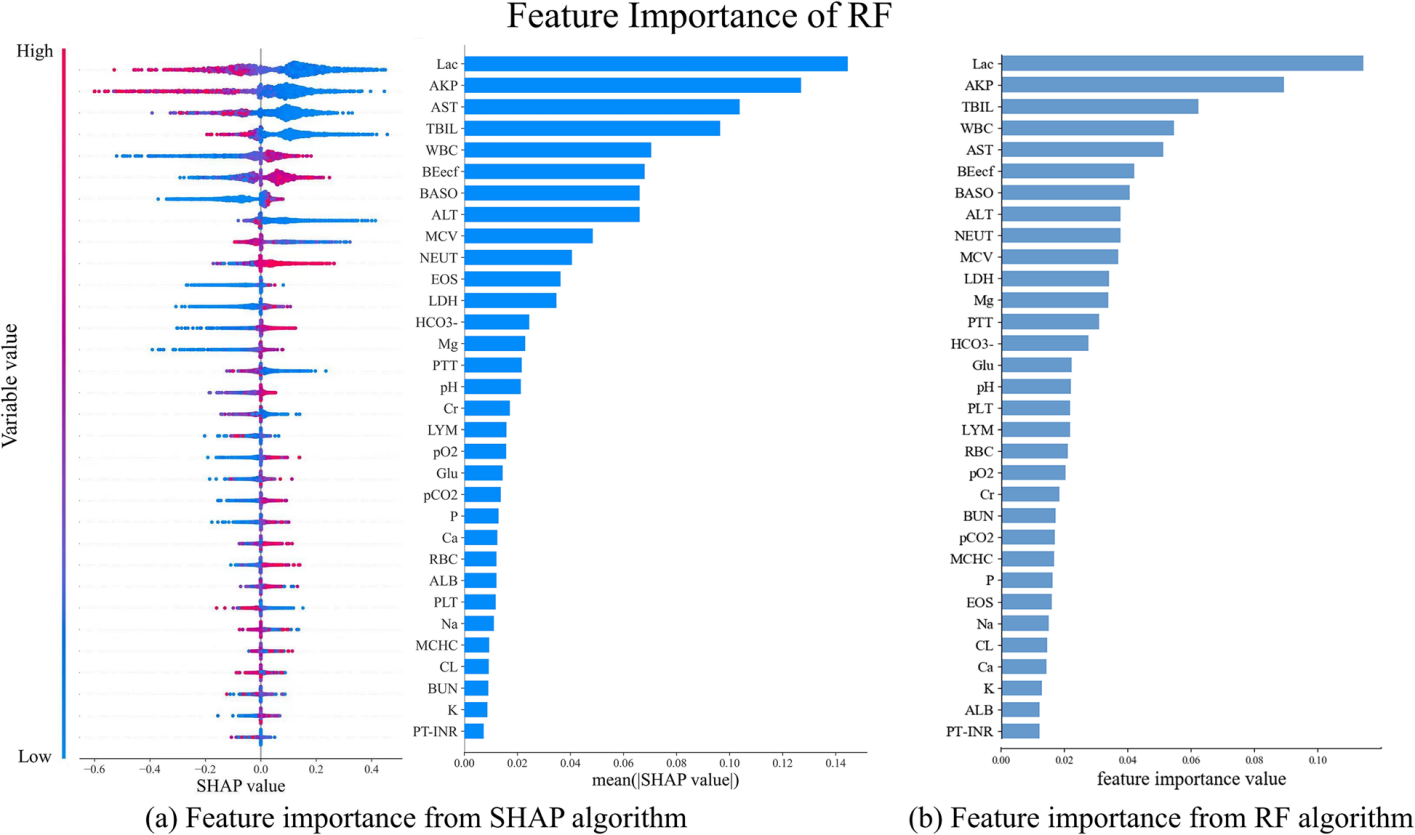
Importance of the variables in the model based on RF. **a** The left plot presents the SHAP value for each variable, with each point representing an individual sample. The vertical axis and horizontal axis represent the variables and the SHAP values, respectively. The color of each point represents the value of the variables, as shown in the color bar on the left. The SHAP values are directly associated with the model’s output. The right plot shows the mean of the absolute SHAP values for each variable as an indicator of the importance of the variable. Variables with higher values are thought to be more important. **b** The plot shows the mean of Gini index for each variable as an indicator of the importance of the variable. Variables with higher values are thought to be more important

In order to identify the most important components in the predictive model, SHAP values were calculated for each variable. Figure 6 shows the variables’ importance in the model based on RF. The six most important variables were lactate (Lac), alkaline phosphatase (AKP), asparate aminotransferase (AST), total bilirubin (TBIL), white blood cell count (WBC), and base excess in extracellular fluid (BEecf): gram-positive bacteremia was associated with lower values of Lac, AKP, AST, and TBIL and higher values of WBC and BEecf. Among the six variables, Lac emerged as the most important one, as indicated in Fig. 6a. RF can also provide feature importance, as indicated in Fig. 6b. The feature importance obtained from RF is almost consistent with that computed using SHAP.

**Fig. 7:**
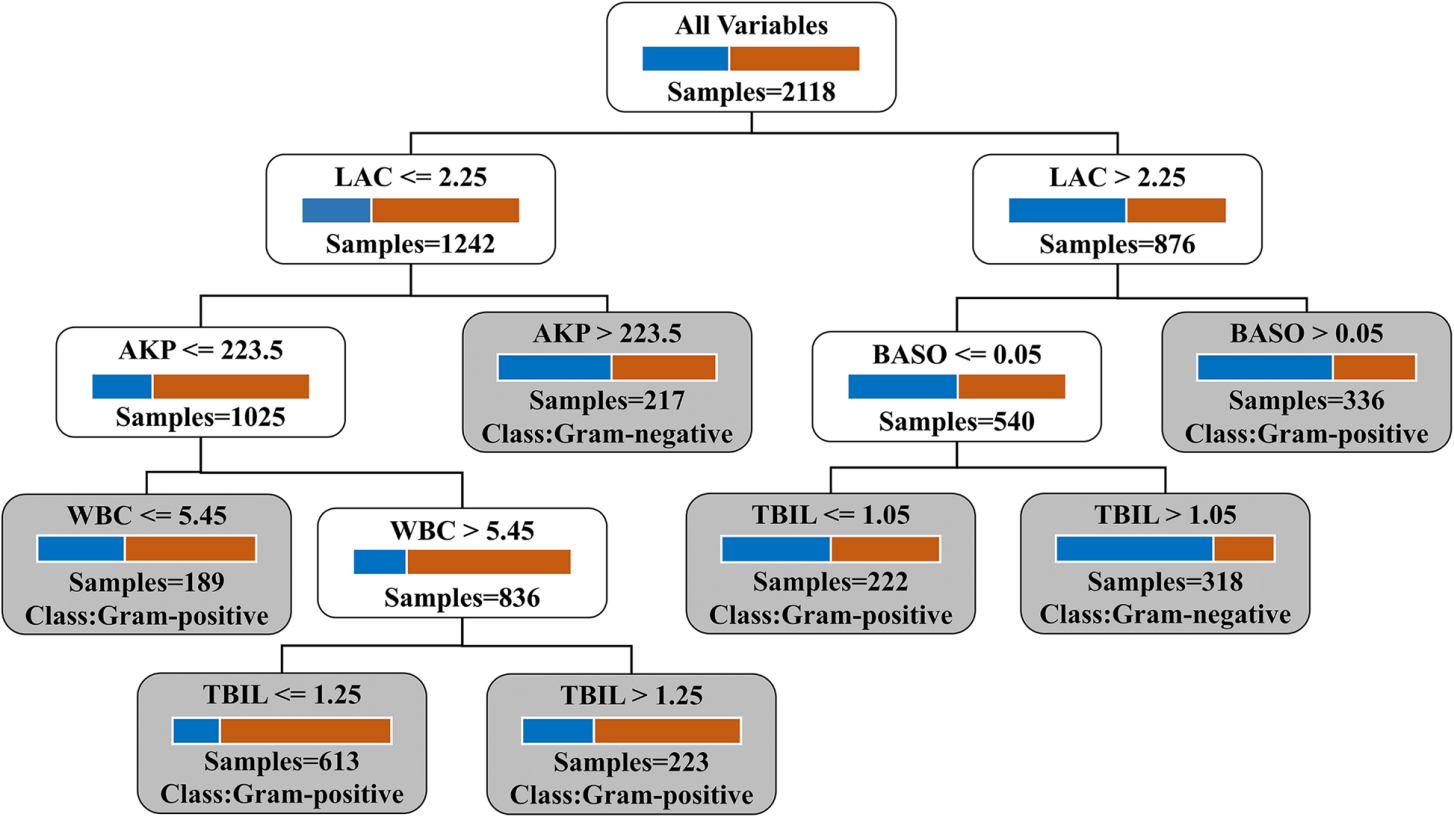
Decision tree for the prediction of gram-positive and gram-negative bacteremia. Blue squares: gram-negative samples, brown squares: gram-positive samples, and gray: leaf nodes. “Samples” refers to the number of samples in the current node. “Class” in the leaf nodes refers to the prediction of the built decision tree, which depends on the number of gram-positive and gram-negative samples. If the number of gram-positive samples is higher than the number of gram-negative samples in the leaf, then the prediction is gram-positive bacteremia

The ten most important variables selected from the RF model (shown in Fig. 6) were used as optional features to build a DT model. Cross-validation was used to select the optimal variable set for DT. Finally, only five variables were selected, as shown in Fig. 7: WBC count, percentage of basophils, alkaline phosphatase, lactate, and total bilirubin. The AUC of the built tree was 0.679 (95% CI = 0.632–0.723), and it had an accuracy of 66.75%, sensitivity of 66%, specificity of 67.82%, PPV of 74.66%, and NPV of 58.13%.

### Evaluation of the RF model in the Qilu Hospital cohort

**Fig. 8:**
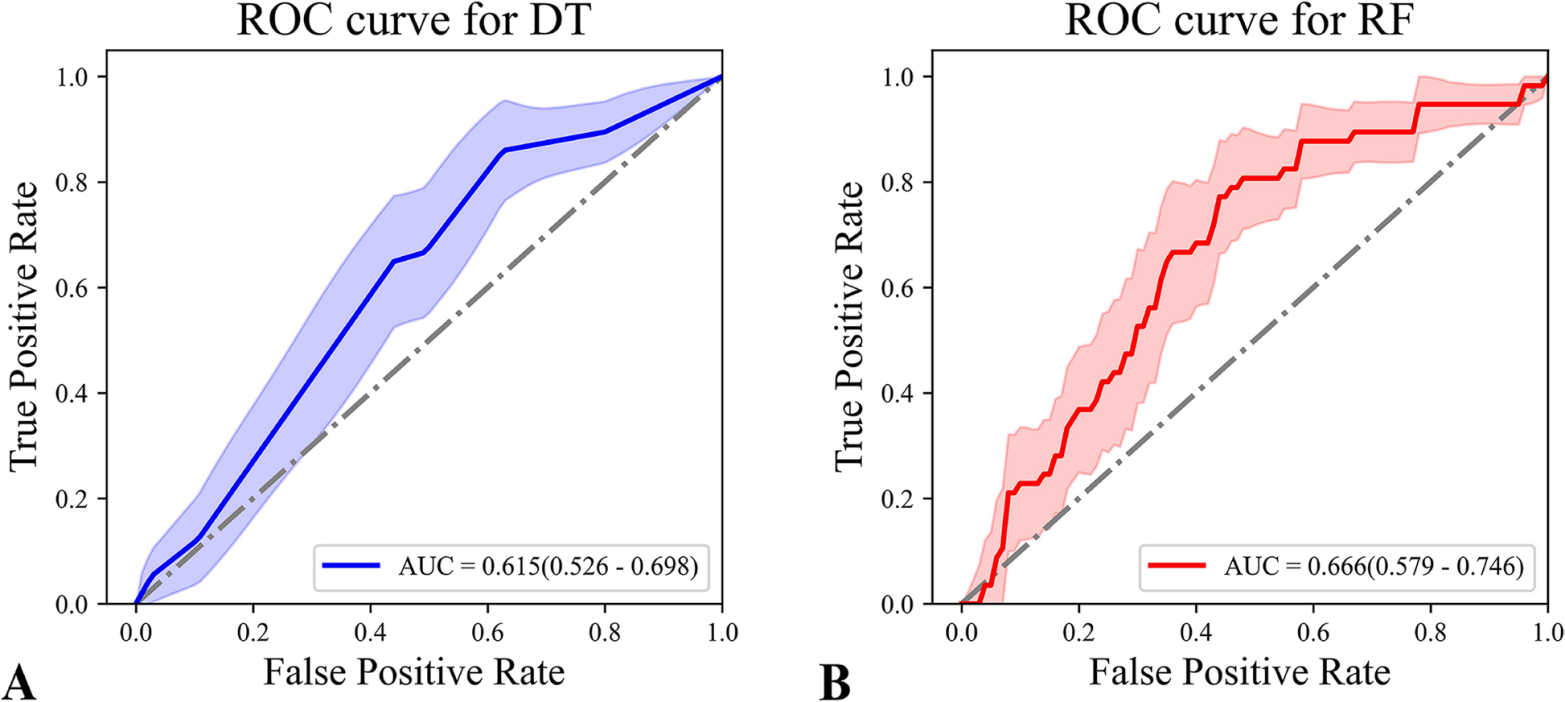
ROC curves of the proposed predictive model based on RF (**A**) and DT (**B**) in the Qilu Hospital cohort

An independent test cohort of 132 critical care patients was recruited from Qilu Hospital of Shandong University to evaluate the performance of the models. The patients’ characteristics and laboratory parameters are shown in Supplementary Table S1. Table 2 shows the performances of the proposed model based on RF and DT in the Qilu Hospital cohort, and Fig. 8 shows the ROC curves for the model. RF (accuracy = 64.39%, AUC = 0.666) outperformed DT (accuracy = 59.85%, AUC = 0.615). Although the performance of the models based on RF and DT decreased to a certain extent in the Qilu hospital cohort compared to the MIMIC dataset, both classifiers still showed acceptable performance in terms of predicting gram-positive and gram-negative bacteremia.

**Table 2:** The performances of the proposed model and DT in Qilu Hospital dataset

	Accuracy	Sensitivity	Specificity	PPV	NPV
RF	64.39%	63.16%	65.33%	58.06%	70.00%
DT	59.85%	64.91%	56.00%	52.86%	67.74%

A software was developed to make the RF- and DT-based prediction models easily accessible (we can provide the software if needed). Figure S1 shows the interface of the software: the input includes two parts that can function independently. Quick Prediction refers to the DT-based model, for which five input variables are required, while General Prediction refers to the RF-based model, which requires more input variables but provides a more precise prediction.

## Discussion

The aim of the current study was to develop a predictive model based on ML algorithms to discriminate between gram-positive and gram-negative bacteremia in patients with severe bloodstream infection before the pathogenic test results are accessible. The model based on the RF algorithm showed satisfactory predictive performance in discriminating between gram-positive and gram-negative pathogens that cause bacteremia. In order to improve its applicability in real-life situations in which all routine blood test results might not be available on time, especially in emergency situations, a DT model was built using only five variables.

Empiric antimicrobial treatment of bacteremia is often problematic because of the increasing resistance of both gram-positive and gram-negative microbes against antimicrobial drugs. Gram-positive bacteria are a major concern, especially multidrug-resistant bacteria such as methicillin-resistant *Staphylococcus Aureus*, vancomycin-resistant *Enterococcus faecium*, and β-lactamase-resistant *Streptococcus pneumonia* [24, 25]. Further, multidrug-resistant gram-negative bacteria, such as Enterobacteriaceae, *Pseudomonas aeruginosa*, and *Acinetobacter baumannii*, also pose a serious and rapidly emerging threat, especially for patients in intensive care units [26]. The easy-to-use model proposed in the present study can be used to promptly predict gram-positive and gram-negative bacteremia and could contribute to the timely and adequate elimination of the implicated pathogen. Adequate empiric antimicrobial treatment for sepsis has been demonstrated to directly affect the mortality rate in the ICU [27]. With this model, the probability of gram-positive or gram-negative bacteremia can be calculated offline when the values of the 32 variables are input into the software provided. Further interventional studies based on this prediction model are necessary to verify its effectiveness based on patient outcomes.

Several laboratory blood test parameters have been proposed as potential predictive markers for the discrimination of gram-positive and gram-negative bacterial infections, and these are used to tailor empiric antimicrobial therapy before the results of the pathogen tests are obtained [6, 9, 28, 29]. However, there is no strong evidence for the ability of any of these parameters to predict the infection pathogens. The ML algorithm has been proved to be helpful in combining several variables to discriminate different subsets of patients. So far, there is no ideal ML model for predicting the pathogens that cause bacteremia. The ML model of Ratzinger et al. based on the K-star algorithm had a sensitivity of only 44.6% for detecting gram-negative bacteremia [30]. Although the AUC of their model (0.675) was comparable to that of the present study, it had poor sensitivity (44.6%) and specificity (79.8%). Ratzinger’s research also started with variables from routine laboratory tests, such as CBC count, liver function test, renal function test, serum electrolytes, and coagulation function test, but only seven variables (gender, count of lymphocytes, count of monocytes, percentage of monocytes, fibrinogen, creatinine, and C-reactive protein) were included in the final K-Star model. When building the current RF model, the results of blood gas analysis were also included. Moreover, 32 variables were entered into the RF model. The larger cohort of patients, the higher number of input variables, and the different algorithms used may explain why our model performed better.

Considering that measurements of the 32 variables input in the RF model may not be available in some areas, medical institutions, and units, a well-performing DT model was also constructed with only five routinely measured variables: WBC count, basophil percentage, alkaline phosphatase, and lactate. Gram-negative bacteremia is associated with a higher level of inflammatory response than gram-positive bacteremia [6]. Accordingly, the association of gram-negative bacteremia with increased levels of WBC has also been found in a previous report [31]. Additionally, as basophils are a type of WBC, the inclusion of basophil percentage as an indicator also makes sense. Gram-positive and gram-negative bacteria activate different receptor pathways [32] and cytokine production patterns in the host [33]. Certain cytokines (such as IL-3, IL-5, and GM-CSF) induced by gram-positive bacteria appear to be important developmental factors for basophils [34]. Further, lipopolysaccharide is found in abundance in the outer membrane of most gram-negative bacteria and plays a key role in host–pathogen interaction [35] by increasing lactatemia via enhanced glycolysis [36] and lactate production [35], as well as early and severe impairment of lactate clearance [37]. Furthermore, it causes hepatoxicity by induction of oxidative stress and consequent oxidative damage to biomolecules [38]. These functions of lipopolysaccharide may explain the significant increase in lactate levels and hepatic biomarkers (e.g., AKP and total bilirubin) in patients with gram-negative bacteremia.

Several limitations of this study must be considered. First, the laboratory blood test variables in the MIMIC database do not represent all commonly used infection-related parameters; for example, procalcitonin and C-reactive protein are not reported in the MIMIC database. Further, immune-related parameters, such as CD4, CD8, and HLA-DR, were rarely recorded in the MIMIC database and could not be included when developing the ML model. The exclusion of these parameters may limit the effectiveness of the ML algorithm. Second, blood cultures present varying degrees of false-negative rates, dependent on the specimen acquisition time, usage of antibiotics, and microbial culture techniques, etc. This ML prediction model was based on blood culture results, which may cause bias because the BSI patients with false-negative blood culture results were not included in the datasets. Third, there was limit data of antimicrobial resistance which could be used for machine learning models training or validation. Our model wasn’t helpful to predict the existence of antimicrobial resistance. Fourth, as the datasets were evaluated retrospectively, most of the laboratory blood test results were not obtained on the same day that bacteremia was suspected. As there is no standard turnaround time for laboratory test results, the applicability of the model may be limited in certain situations. Finally, the model needs to be evaluated using data from different regions and countries, as well as prospective cohorts.

## Conclusion

The present ML-based models could effectively discriminate between gram-positive and gram-negative bacteremia based on routine laboratory blood test results. This simple model would be beneficial in terms of guiding timely antibiotic selection and administration in critically ill patients with bacteremia before their pathogen test results are available. This model would be especially useful for patients in developing countries or those admitted to lower-level healthcare centers.

### Supplementary Information

**Additional file 1: Table S1.** Distribution of patients’ demographics characteristics and routine laboratory parameters in Qilu Hospital cohort.

**Additional file 2: Figure S1.** Interface of the prediction system based on the RF and DT models. A: Prediction interface based on Quick Prediction (for the DT model); B: prediction interface based on General Prediction (for the RF model).

## Data Availability

The datasets used during the current study are available from the corresponding author on reasonable request.

## Abbreviations

ML: Machine Learning
MIMIC: Medical Information Mart for Intensive Care
LR: Logistic Regression
SVM: Support Vector Machine
ANN: Artificial Neural Network
RF: Random Forest
CNN: Convolutional Neural Network
ROC: Receiver Operating Characteristic Curve
AUC: Area Under the ROC Curve
DT: Decision Tree
IL: Interleukin
CLABSIs: Central Line-associated Bloodstream Infections
PPV: Positive Predictive Value
NPV: Negative Predictive Value
SHAP: SHapley Additive Explanation
ICU: Intensive Care Unit
PLT: Platelet Count
WBC: White Blood Cell Count
BASO: Basophils
EOS: Eosinophils
LYM: Lymphocytes
NEU: Neutrophils
RBC: Red Blood Cell Count
MCH: Mean Corpuscular Haemoglobin
MCHC: Mean Corpuscular Haemoglobin Concentration
MCV: Mean Corpuscular Volume
AST: Asparate Aminotransferase
ALT: Alanine Aminotransferase
TBIL: Total Bilirubin
AKP: Alkaline Phosphatase
ALB: Albumin
LDH: Lactate Dehydrogenase
Cr: Creatinine
BUN: Blood Urea Nitrogen
Glu: Glucose
Lac: Lactate
BEecf: Base Excess in Extracellular Fluid
pO2: Partial Pressure of Arterial Oxygen
pCO2: Partial Pressure of Arterial Carbon Dioxide
PT-INR: Prothrombin Time-International Normalized Ratio
PTT: Partial Thromboplastin Time
CL: Chloride
K: Potassium
Na: Sodium
Mg: Magnesium
Ca: Calcium
P: Phosphate
HCO3^−^: Bicarbonate
MDR: Multidrug-resistant
MRSA: Methicillin-resistant Staphylococcus Aureus
VRE: Vancomycin-resistant Enterococcus Faecium
GBM: Gradient Boosting Machine

## References

[1] Timsit JF, Ruppe E, Barbier F, Tabah A, Bassetti M (2020). Bloodstream infections in critically ill patients: an expert statement. Intensive Care Med.

[2] Adrie C, Garrouste-Orgeas M, Ibn Essaied W, Schwebel C, Darmon M, Mourvillier B (2017). Attributable mortality of ICU-acquired bloodstream infections: Impact of the source, causative micro-organism, resistance profile and antimicrobial therapy. J Infect.

[3] Zahar JR, Timsit JF, Garrouste-Orgeas M, Francais A, Vesin A, Descorps-Declere A (2011). Outcomes in severe sepsis and patients with septic shock: pathogen species and infection sites are not associated with mortality. Crit Care Med.

[4] Pouwels KB, Vansteelandt S, Batra R, Edgeworth JD, Smieszek T, Robotham JV (2018). Intensive care unit (ICU)-acquired bacteraemia and ICU mortality and discharge: addressing time-varying confounding using appropriate methodology. J Hosp Infect.

[5] Kumar A, Ellis P, Arabi Y, Roberts D, Light B, Parrillo JE (2009). Initiation of inappropriate antimicrobial therapy results in a fivefold reduction of survival in human septic shock. Chest.

[6] Abe R, Oda S, Sadahiro T, Nakamura M, Hirayama Y, Tateishi Y (2010). Gram-negative bacteremia induces greater magnitude of inflammatory response than Gram-positive bacteremia. Crit Care.

[7] Bilgili B, Haliloglu M, Aslan MS, Sayan I, Kasapoglu US, Cinel I (2018). Diagnostic accuracy of procalcitonin for differentiating bacteraemic gram-negative sepsis from gram-positive sepsis. Turk J Anaesthesiol Reanim.

[8] Liu HH, Zhang MW, Guo JB, Li J, Su L (2017). Procalcitonin and C-reactive protein in early diagnosis of sepsis caused by either gram-negative or gram-positive bacteria. Ir J Med Sci.

[9] Xu XJ, Tang YM, Liao C, Song H, Yang SL, Xu WQ (2013). Inflammatory cytokine measurement quickly discriminates gram-negative from gram-positive bacteremia in pediatric hematology/oncology patients with septic shock. Intensive Care Med.

[10] Debas HT, Donkor P, Gawande A, Jamison DT, Kruk ME, Mock CN, editors. 2015. Essential Surgery. Disease Control Priorities, third edition, volume 1. Washington, DC: World Bank. 10.1596/978-1-4648-0346-8. 26740991

[11] Richens JG, Lee CM, Johri S (2020). Improving the accuracy of medical diagnosis with causal machine learning. Nat Commun.

[12] Lynch CJ, Liston C (2018). New machine-learning technologies for computer-aided diagnosis. Nat Med.

[13] Beeler C, Dbeibo L, Kelley K, Thatcher L, Webb D, Bah A (2018). Assessing patient risk of central line-associated bacteremia via machine learning. Am J Infect Control.

[14] Mahmoud E, Al Dhoayan M, Bosaeed M, Al Johani S, Arabi YM (2021). Developing machine-learning prediction algorithm for bacteremia in admitted patients. Infection and drug resistance.

[15] Tsai CM, Lin CR, Zhang H, Chiu IM, Cheng CY, Yu HR (2020). Using machine learning to predict bacteremia in febrile children presented to the Emergency Department. Diagnostics (Basel, Switzerland).

[16] Bhavani SV, Lonjers Z, Carey KA, Afshar M, Gilbert ER, Shah NS (2020). The development and validation of a machine learning model to predict bacteremia and fungemia in hospitalized patients using electronic health record data. Crit Care Med.

[17] Johnson AEW, Pollard TJ, Shen L, Lehman L-wH, Feng M, Ghassemi M (2016). MIMIC-III, a freely accessible critical care database. Scientific Data.

[18] Johnson Alistair BL, Pollard Tom, Horng Steven, Celi Leo Anthony, Roger Mark. MIMIC-IV (version 1.0). PhysioNet.2021. 10.13026/s6n6-xd98.

[19] Goldberger AL, Amaral LA, Glass L, Hausdorff JM, Ivanov PC, Mark RG (2000). PhysioBank, PhysioToolkit, and PhysioNet: components of a new research resource for complex physiologic signals. Circulation.

[20] Hall KK, Lyman JA (2006). Updated review of blood culture contamination. Clin Microbiol Rev.

[21] Weinstein MP, Towns ML, Quartey SM, Mirrett S, Reimer LG, Parmigiani G (1997). The clinical significance of positive blood cultures in the 1990s: a prospective comprehensive evaluation of the microbiology, epidemiology, and outcome of bacteremia and fungemia in adults. Clin Infect Dis.

[22] Muehlematter UJ, Mannil M, Becker AS, Vokinger KN, Finkenstaedt T, Osterhoff G (2019). Vertebral body insufficiency fractures: detection of vertebrae at risk on standard CT images using texture analysis and machine learning. Eur Radiol.

[23] Lundberg SM, Erion, Gabriel G, Lee SI. Consistent individualized feature attribution for tree ensembles. eprint arXiv:180203888. 2018. 10.48550/arXiv.1802.03888.

[24] Cornaglia G (2009). Fighting infections due to multidrug-resistant gram-positive pathogens. Clin Microbiol Infect.

[25] Asokan GV, Ramadhan T, Ahmed E, Sanad H (2019). WHO global priority pathogens list: a bibliometric analysis of Medline-Pubmed for knowledge mobilization to infection prevention and control practices in Bahrain. Oman Med J.

[26] Cerceo E, Deitelzweig SB, Sherman BM, Amin AN (2016). Multidrug-resistant gram-negative bacterial infections in the hospital setting: overview, implications for clinical practice, and emerging treatment options. Microb Drug Resist.

[27] Garnacho-Montero J, Garcia-Garmendia JL, Barrero-Almodovar A, Jimenez-Jimenez FJ, Perez-Paredes C, Ortiz-Leyba C (2003). Impact of adequate empirical antibiotic therapy on the outcome of patients admitted to the intensive care unit with sepsis. Crit Care Med.

[28] Chase M, Klasco RS, Joyce NR, Donnino MW, Wolfe RE, Shapiro NI (2012). Predictors of bacteremia in emergency department patients with suspected infection. Am J Emerg Med.

[29] Brodská H, Malíčková K, Adámková V, Benáková H, Šťastná MM, Zima T (2013). Significantly higher procalcitonin levels could differentiate gram-negative sepsis from gram-positive and fungal sepsis. Clin Exp Med.

[30] Ratzinger F, Dedeyan M, Rammerstorfer M, Perkmann T, Burgmann H, Makristathis A (2015). Neither single nor a combination of routine laboratory parameters can discriminate between gram-positive and gram-negative bacteremia. Sci Rep.

[31] Vandijck DM, Hoste EA, Blot SI, Depuydt PO, Peleman RA, Decruyenaere JM (2007). Dynamics of C-reactive protein and white blood cell count in critically ill patients with nosocomial gram positive vs. gram negative bacteremia: a historical cohort study. BMC Infect Dis.

[32] Hoerr V, Zbytnuik L, Leger C, Tam PP, Kubes P, Vogel HJ (2012). Gram-negative and Gram-positive bacterial infections give rise to a different metabolic response in a mouse model. J Proteome Res.

[33] Karlsson H, Larsson P, Wold AE, Rudin A (2004). Pattern of cytokine responses to gram-positive and gram-negative commensal bacteria is profoundly changed when monocytes differentiate into dendritic cells. Infect Immun.

[34] Sarmiento EU, Espiritu BR, Gleich GJ, Thomas LL (1995). IL-3, IL-5, and granulocyte-macrophage colony-stimulating factor potentiate basophil mediator release stimulated by eosinophil granule major basic protein. J Immunol.

[35] Michaeli B, Martinez A, Revelly JP, Cayeux MC, Chiolero RL, Tappy L (2012). Effects of endotoxin on lactate metabolism in humans. Crit Care.

[36] Khatib-Massalha E, Bhattacharya S, Massalha H, Biram A, Golan K, Kollet O (2020). Lactate released by inflammatory bone marrow neutrophils induces their mobilization via endothelial GPR81 signaling. Nat Commun.

[37] Tapia P, Soto D, Bruhn A, Alegria L, Jarufe N, Luengo C (2015). Impairment of exogenous lactate clearance in experimental hyperdynamic septic shock is not related to total liver hypoperfusion. Crit Care.

[38] Jirillo E, Caccavo D, Magrone T, Piccigallo E, Amati L, Lembo A (2002). The role of the liver in the response to LPS: experimental and clinical findings. J Endotoxin Res.

