# Supplementary material for: Machine learning model for the prediction of gram-positive and gram-negative bacterial bloodstream infection based on routine laboratory parameters
Source: BMC Infect Dis. 2023 Oct 10;23:675. doi: 10.1186/s12879-023-08602-4 (PMC10566101; doi:10.1186/s12879-023-08602-4)
Supplement: Supplementary file 1 — Additional file 1: Table S1. Distribution of patients’ demographics characteristics and routine laboratory parameters in Qilu Hospital cohort. [file 12879_2023_8602_MOESM1_ESM.docx]

**Table S1 Distribution of patients’ demographics characteristics and routine laboratory parameters in Qilu Hospital cohort**

| **Variables** | **Total** | **Gram-negative** | **Gram-positive** |
| --- | --- | --- | --- |
| PLT, mean(SD), K/uL | 222.14(550.9) | 166.75(167.59) | 295.04(814.44) |
| WBC, mean(SD), K/uL | 12.42(7.65) | 13.31(7.73) | 11.25(7.44) |
| MCHC, mean(SD), % | 31.84(1.39) | 31.94(1.44) | 31.71(1.33) |
| MCV, mean(SD), fL | 94.75(6.09) | 93.91(6.43) | 95.86(5.48) |
| RBC, mean(SD), m/uL | 2.85(0.68) | 2.78(0.71) | 2.94(0.65) |
| Cr, mean(SD), mg/dL | 1.45(1.09) | 1.53(1.11) | 1.34(1.06) |
| CL, mean(SD), mEq/L | 107.37(8.24) | 106.4(7.49) | 108.65(9.04) |
| K, mean(SD), mEq/L | 4.38(0.64) | 4.34(0.67) | 4.43(0.61) |
| Na, mean(SD), mEq/L | 145.77(10.22) | 145.25(10.31) | 146.46(10.15) |
| BUN, mean(SD), mg/dL | 48.35(32.31) | 50.7(31.78) | 45.25(33.02) |
| HCO3-, mean(SD), mEq/L | 26.07(5.88) | 25.42(5.71) | 26.93(6.05) |
| Glu, mean(SD), mg/dL | 151.57(57.89) | 155.21(57.56) | 146.78(58.48) |
| Mg, mean(SD), mg/dL | 2.15(0.55) | 2.08(0.51) | 2.25(0.59) |
| Ca, mean(SD), mg/dL | 8.52(0.82) | 8.46(0.78) | 8.59(0.86) |
| P, mean(SD), mg/dL | 3.01(1.52) | 3.05(1.73) | 2.95(1.2) |
| PT-INR, mean(SD) | 1.41(0.73) | 1.42(0.6) | 1.4(0.89) |
| PTT, mean(SD), sec | 40.78(22.44) | 43.02(23.81) | 37.83(20.33) |
| AST, mean(SD), IU/L | 106.02(297.33) | 136.89(380.52) | 65.39(111.87) |
| ALT, mean(SD), IU/L | 74.8(169.99) | 74.0(180.97) | 75.86(155.93) |
| TBIL, mean(SD), mg/dL | 2.56(4.43) | 3.05(4.98) | 1.9(3.53) |
| AKP, mean(SD), IU/L | 152.65(107.79) | 180.59(127.55) | 115.89(57.11) |
| BASO, mean(SD), % | 0.08(0.28) | 0.07(0.16) | 0.09(0.39) |
| EOS, mean(SD), % | 0.15(0.28) | 0.17(0.35) | 0.12(0.12) |
| LYM, mean(SD), % | 11.71(8.37) | 11.26(9.62) | 12.29(6.41) |
| Neutrophils, mean(SD), % | 82.57(29.62) | 81.5(27.26) | 83.98(32.65) |
| Albumin, mean(SD), g/dL | 3.26(0.6) | 3.18(0.61) | 3.37(0.56) |
| Lac, mean(SD), mmol/L | 3.44(4.97) | 3.72(4.8) | 3.06(5.2) |
| LDH, mean(SD), IU/L | 566.06(859.49) | 546.29(768.49) | 592.07(972.86) |
| pH, mean(SD), units | 7.42(0.11) | 7.4(0.11) | 7.45(0.09) |
| BEecf, mean(SD), mEq/L | 1.59(6.94) | 0.57(6.75) | 2.92(7.0) |
| pO2, mean(SD), mm Hg | 98.4(42.16) | 88.83(34.69) | 111.0(47.78) |
| pCO2, mean(SD), mm Hg | 40.14(11.47) | 41.32(13.31) | 38.58(8.31) |
