# Supplementary material for: Machine learning model for the prediction of gram-positive and gram-negative bacterial bloodstream infection based on routine laboratory parameters
Source: BMC Infect Dis. 2023 Oct 10;23:675. doi: 10.1186/s12879-023-08602-4 (PMC10566101; doi:10.1186/s12879-023-08602-4)
Supplement: Supplementary file 2 — Additional file 2: Figure S1. Interface of the prediction system based on the RF and DT models. A: Prediction interface based on Quick Prediction (for the DT model); B: prediction interface based on General Prediction (for the RF model). [file 12879_2023_8602_MOESM2_ESM.docx]

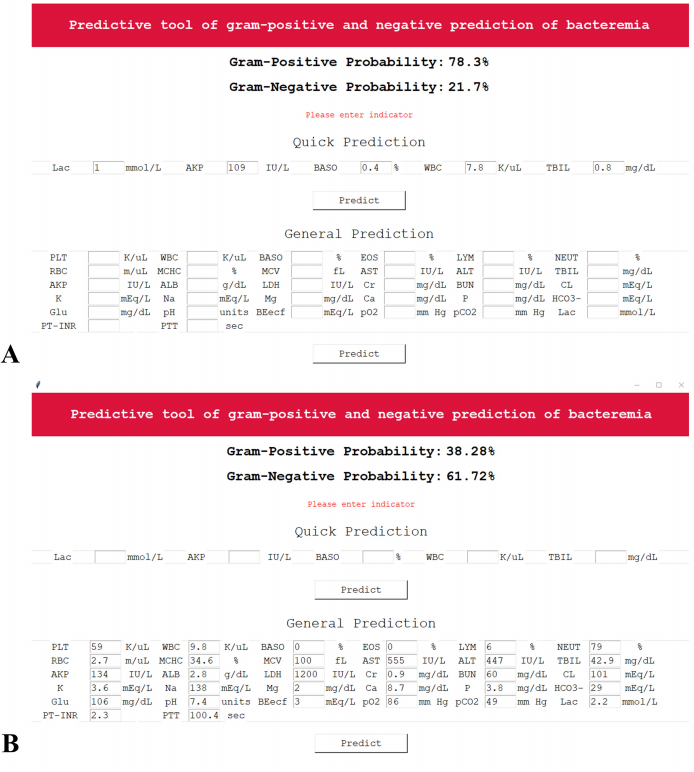


**Figure S1** Interface of the prediction system based on the RF and DT models. A: Prediction interface based on Quick Prediction (for the DT model); B: prediction interface based on General Prediction (for the RF model).
